# The Role of Ion Channels in Cervical Cancer Progression: From Molecular Biomarkers to Diagnostic and Therapeutic Innovations

**DOI:** 10.3390/cancers17091538

**Published:** 2025-05-01

**Authors:** Elżbieta Bartoszewska, Melania Czapla, Katarzyna Rakoczy, Michał Filipski, Katarzyna Rekiel, Izabela Skowron, Julita Kulbacka, Christopher Kobierzycki

**Affiliations:** 1 Faculty of Medicine, Wroclaw Medical University, Mikulicza-Radeckiego 5, 50-345 Wroclaw, Poland; elzbieta.bartoszewska@student.umw.edu.pl (E.B.); melania.czapla@student.umw.edu.pl (M.C.); katarzyna.rakoczy@student.umw.edu.pl (K.R.); michal.filipski@student.umw.edu.pl (M.F.); katarzyna.rekiel@student.umw.edu.pl (K.R.); izabela.skowron@student.umw.edu.pl (I.S.); 2 Student Research Group No. K148, Faculty of Pharmacy, Wroclaw Medical University, Borowska 211A, 50-556 Wroclaw, Poland; 3 Department of Molecular and Cellular Biology, Wroclaw Medical University, Borowska 211A, 50-556 Wroclaw, Poland; 4 Department of Immunology and Bioelectrochemistry, State Research Institute Centre for Innovative Medicine, LT-08406 Vilnius, Lithuania; 5 Division of Histology and Embryology, Department of Human Morphology and Embryology, Wroclaw Medical University, 50-368 Wroclaw, Poland

**Keywords:** ion channels, cervical cancer, biomarkers, therapeutic innovations

## Abstract

Ion channels are proteins that regulate the flow of ions across cell membranes, playing a vital role in cervical cancer development and progression. These channels serve as both potential diagnostic markers and therapeutic targets, offering new opportunities for cancer treatment. Moreover, ion channels are crucial molecular indicators and possible therapeutic targets due to their role in the development of cervical cancer. Our review focuses on the various types of ion channels which are associated with cervical cancer (CCa), including sodium, calcium, and potassium channels. In our review, we clarify their diagnostic and prognostic value, as well as their relationship to the prognosis and stage of the disease. We also examine how ion channels contribute to the metastasis of cervical cancer, specifically in relation to their influence on cell motility, invasion, and interaction with the tumor microenvironment. By examining preclinical and clinical research involving ion channel blockers and modulators, we also highlight the therapeutic potential of targeting ion channels. We have demonstrated the available assays and imaging methods based on ion channel activity as examples of emerging diagnostic breakthroughs that show promise for enhancing the early detection of cervical cancer. Additionally, the possibility that ion channel modulator-based combination therapy could improve the efficacy of traditional treatments is investigated. To demonstrate the potential of ion channels in cervical cancer diagnosis and treatment, our review highlights the current challenges and the promising role in cervical cancer diagnostics and therapy.

## Simple Summary

Cervical cancer remains one of the most serious health challenges for women worldwide, causing 350,000 deaths in 2022 despite advances in prevention through vaccination and screening. This study focuses on understanding how tiny gateways in cell walls, called ion channels, influence cervical cancer development and treatment. These channels, which act like doors that control the movement of essential particles in and out of cells, can behave abnormally in cancer cells, helping the disease grow and spread. Our research explores two main possibilities: using these channels as early warning signs to detect cervical cancer more accurately, and developing new treatments that target these channels to fight the disease. We found that certain drugs that affect these channels, especially when combined with standard cancer treatments, show promise in stopping cancer growth and improving treatment effectiveness. This research is particularly important for developing countries, where current screening methods may be harder to access. While more studies are needed to develop practical applications, our findings suggest that targeting ion channels could lead to better diagnostic tools and more effective treatments, potentially helping save many lives worldwide.

## 1. Introduction

### 1.1. The Role of Ion Channels in Cellular Physiology

Ion channels are integral membrane proteins that facilitate the selective passage of ions, such as sodium (Na^+^), potassium (K^+^), calcium (Ca^2+^), and chloride (Cl^−^), across cellular membranes. Ion channels play critical roles in a variety of biological functions, including nerve signal transmission, heart contraction, temperature sensing, hormone release, and apoptotic control [1]. Ion channels play an essential role in cell growth and homeostasis [2]. They are essential in establishing and maintaining the resting membrane potential of cells. Potassium channels allow K^+^ ions to flow out of the cell, providing a negative charge inside the cell compared to the outside, creating an electrochemical gradient [3].

Another essential function is the regulation of intracellular calcium levels. Cells control calcium concentrations in distinct cellular compartments via a variety of ion channels, pumps, and transporters situated at the plasma membrane’s surface and interior organelles [4]. This involves calcium ion channels, such as those involved in store-operated calcium entry (SOCE) [5]. The regulation of intracellular Ca^2+^ concentrations is critical for various cellular functions, including muscle contraction, secretion, and gene expression [5]. In addition to being common messengers of biological processes, calcium ions influence long-term metabolic and transcriptional cell reprogramming, which controls cell proliferation, differentiation, and migration [4].

Ion channels play a pivotal role in signal transduction by allowing the flow of ions that act as secondary messengers in various signaling pathways. Cells produce ion channels accessible to ions, such as calcium, potassium, sodium, and chloride, which control the potential of electricity across biological membranes, allowing for finer control over the initiation of signaling cascades that regulate cellular responses to external stimuli [4]. Furthermore, mechanosensitive ion channels, such as PIEZO1, regulate cell volume, preventing cellular swelling or shrinkage [6].

### 1.2. Tumorigenesis Mechanisms Influenced by Ion Channels

**Figure 1:**
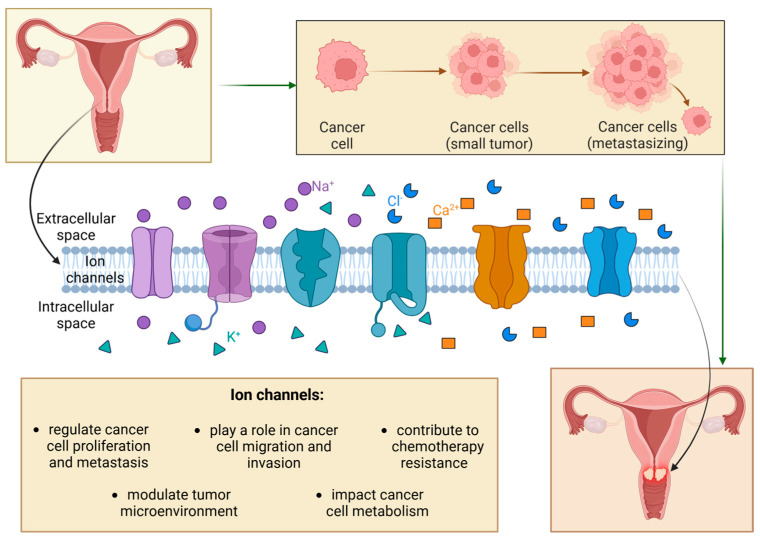
The role of ion channels in cervical cancer tumorigenesis.

The gain or loss of function of ion channels, caused by mutations or changes in regulation, expression, or oligomerization in biological membranes, disrupts the homeostasis of ion channels and alters the ion signaling network [4]. Those alterations can lead to various diseases, including neurodegenerative disorders and neoplasms [7]. Ion channels play crucial regulatory roles in cancer pathophysiology, performing several functions from the initial transformation of cancers to metastasis. Heterogeneous ion channel distribution is associated with distinct phases of cancer, such as cell proliferation, metastasis, dissemination, and apoptosis [1]. Cancer cells use ion channels to promote their progress, expand as tumors, and integrate into a microenvironment, including various non-cancerous cells (Figure 1) [2].

Many ion channels, especially calcium channels like SOCE and voltage-gated calcium channels, regulate intracellular calcium signaling pathways. Elevated calcium influx activates PI3K/AKT and MAPK pathways, driving cell cycle progression and causing uncontrolled proliferation. Calcium-dependent enzymes, such as matrix metalloproteinases (MMPs), degrade the extracellular matrix (ECM), facilitating cell migration and metastasis [8]. Moreover, voltage-gated sodium and potassium channels regulate the cancer cell membranes’ electrical and mechanical properties. Sodium channels, such as Nav1.5, increase intracellular calcium, promoting cytoskeletal remodeling and invasiveness. Mechanosensitive channels respond to extracellular forces, enhancing metastatic potential [9].

Ion channels alter ionic balance and extracellular pH, creating a microenvironment conducive to tumor growth. Hypoxia-induced sodium/hydrogen exchangers and chloride channels lead to acidification, enhancing angiogenesis and ECM degradation [10]. Mechanosensitive ion channels, like PIEZO1, further respond to tumor mechanical cues, regulating angiogenesis and cell migration [11]. A higher concentration of hormones may also cause an increased distribution of ion channels, which leads to cancer cell proliferation [2].

Another significant ability of proteins discussed in this paper is their impact on cancer cell metabolism. Potassium channels maintain membrane potential, which is crucial for ATP synthesis in mitochondria. Dysregulation of sodium and chloride channels affects ionic gradients, influencing glycolysis and oxidative phosphorylation. These metabolic changes allow cancer cells to thrive in hostile microenvironments, including nutrient-deprived and hypoxic conditions, promoting tumor survival [12]. Moreover, the volume-sensitive chloride channels display osmosensing abilities. Through various interactions, they are able to regulate vital cell processes, affecting the course of the disease [13]. Additionally, ion channels promote chemotherapy resistance by activating survival pathways and efflux pumps.

Overexpression of the potassium channels hyperpolarizes the membrane, reducing drug uptake. Calcium and sodium channels enhance efflux pump activity, such as P-glycoprotein, and inhibit apoptotic pathways, protecting cancer cells from therapeutic agents [14].

### 1.3. Types of Ion Channels Implicated in Cervical Cancer

Cervical cancer is one of the leading health concerns for today’s females. It ranks as the fourth most frequent disease in women worldwide, with about 660,000 new cases and 350,000 deaths in 2022 [15]. Although vaccination and screening initiatives have lowered the global incidence of CCa by more than 80%, the death prevalence in low-income countries is still high [16]. The primary etiological agent is human papillomavirus. The cytological Pap smear test is the most commonly used procedure for cervical cancer screening. Despite its effectiveness in identifying precancerous lesions and responding to the disease’s early stages, higher screening and diagnostic reliability, as well as increased specificity and sensitivity, are required [17]. That is why new methods of detection and therapy based on cancer pathophysiology are being developed. One of the most promising areas is research on the following ion channels. Table 1 presents the important ion channels and their characteristics.

The aim of this review is to explore the various roles of ion channels in cervical cancer progression. It highlights their significance as molecular biomarkers and therapeutic targets. It examines their involvement in processes such as metastasis, cell migration, and tumor microenvironment interaction, while also discussing diagnostic advancements and the potential of ion channel modulators to improve conventional treatments.

## 2. Ion Channels as Molecular Biomarkers in Cervical Cancer

The process of carcinogenesis in cervical cancer is complex and undoubtedly depends on persistent infection with the human papillomavirus (HPV). However, it is essential to note that even 80% of women who have been exposed to HPV never develop cervical cancer. Therefore, thorough research of the processes in which individual ion channels are involved is crucial for better understanding the disease’s pathogenesis and for creating modern diagnostic and prognostic methods [11,12]. It should also be noted that, just as the process of carcinogenesis depends on various factors, such as viral infections, hormonal influences, or the presence of carcinogens in tobacco smoke, it is equally challenging to demonstrate the direct effect of HPV on gene expression and the function of ion channels [13]. Among many ion channels that are widely studied, potassium, calcium, and sodium channels seem to be the most promising biomarkers for cervical cancer.

### 2.1. Potassium Channels

Potassium channels are one of the most studied in gynecological cancers due to their role in cancer cell proliferation, apoptosis, and migration processes. Several types of potassium channels have been linked to tumorigenesis. They could be potentially used as biomarkers—voltage-gated potassium channels (K_V_), calcium-activated potassium channels (K_Ca_), and ATP-sensitive potassium channels (K_ATP_), in particular [13,14,15].

#### 2.1.1. Voltage-Gated Potassium Channels

The K_V_1.1 channel (encoded by the KCNA1 gene) functions as a potassium-selective channel, allowing the passage of potassium cations according to the electrochemical gradient. However, significantly higher levels of KCNA1 mRNA have been observed in cancer cells compared to healthy tissue, with the highest expression of this gene found in HeLa cells. One study has shown that overexpression promotes cancer cell growth, proliferation, and migration, ultimately increasing the invasive potential of the tumor. It also established the following correlation: the higher the KCNA1 expression, the shorter the predicted survival and the worse the prognosis. Monitoring KCNA1 expression levels might serve as a marker for evaluating cancer progression and estimating survival outcomes [16]. The KCNH1 gene could be another promising biomarker. It encodes the Kv10.1 channel that belongs to the Ether-à-go-go (EAG) superfamily, and while it physiologically occurs in tissues such as the brain, placenta, testes, and adrenal glands, its significantly elevated expression has been observed in cervical cancer tissues. Studies have shown that inhibiting KCNH1 suppresses cell proliferation and limits tumor growth [17]. Although further research is needed, KCNH1 overexpression is suggested to be a useful marker for monitoring tumor progression and potential metastasis [18].

#### 2.1.2. Calcium-Activated Potassium Channels

Among calcium-activated potassium channels, two of them seem to be particularly important for the development of modern diagnostic methods for cervical cancer. Increased expression of the KCNMA1 gene, which encodes the K_Ca_1.1 channel, has been observed both in mice with induced tumors and in cervical biopsy samples. No expression of this gene was detected in non-cancerous tissues, while more than half of LSIL (low-grade squamous intraepithelial lesion) samples showed its presence, and in HSIL (high-grade squamous intraepithelial lesion) and cervical cancer biopsies, KCNMA1 expression was found in all cases [19]. Monitoring KCNMA1 gene expression could provide valuable data on the progression of dysplastic changes. Moreover, the KCa3.1 channel, encoded by the KCNN4 gene, has shown overexpression in cervical cancer tissues and HeLa cell lines. Its expression level is correlated with the advancement of histopathological changes, which emphasizes its potential as a diagnostic marker [20].

#### 2.1.3. ATP-Sensitive Potassium Channels

K-ATP channels physiologically occur in various tissues, such as cardiomyocytes, skeletal and smooth muscle cells, the brain, and the pancreas. These channels are heterooctameric membrane complexes composed of four Kir6.X subunits and four sulfonylurea receptor (SUR) subunits. Studies on cell lines and tumor tissue samples have revealed the presence of Kir6.2, SUR1, SUR2a, and SUR2b subunits, which are not characteristic of healthy cervical tissue. It is worth noting that the role of SUR2 subunits remains a topic of debate in the literature. High levels of Kir6.2 expression were observed in both cell lines and cervical squamous cell carcinoma samples at stage IV, as well as in highly invasive tumors [10,15]. Furthermore, it was found that the E7 oncoprotein significantly upregulates the expression of the ABCC8 gene, which encodes the SUR1 protein, via a positive feedback mechanism. The highest expression levels were detected in CIN III (cervical intraepithelial neoplasia grade III) samples and cervical squamous cell carcinoma cases, suggesting that K-ATP channels may serve as an additional marker for dysplasia progression and malignancy [10].

### 2.2. Calcium Channels

Calcium channels have recently gained significant interest in molecular diagnostics, particularly TRP (transient receptor potential channels) and CRAC (calcium release-activated calcium channels) families, which are characterized the most. Studies have shown that alterations in the expression of specific proteins and channels affect the processes of cancer cell proliferation and their potential for tissue invasion and metastasis. Furthermore, it was found that some of these channels could be a valuable source of information on patient prognosis [6,21].

#### 2.2.1. Transient Receptor Potential Channels

The TRP superfamily, which includes TRPC, TRPV, and TRPM channels, plays a substantial role in the development of advanced diagnostic methods. One study has demonstrated an increased expression of the gene encoding the TRPC6 channel in HeLa and SiHa cell lines. Additionally, an important correlation was observed between high expression of this gene and tumor invasion of vascular and lymphatic spaces, highlighting its potential in diagnostics and assessment of progression [22]. Among the TRPV subfamily, the TRPV1 channel has been identified as a potential biomarker, although the results of different studies are inconsistent. According to Wang and their research team, TRPV1 expression in squamous cell carcinoma was significantly lower compared to healthy tissue. Their studies indicated that this channel plays a role in processes such as cell differentiation, ferroptosis, inflammatory response, and metabolic regulation. Altered TRPV1 expression may contribute to immune evasion by the tumor. Furthermore, patients with low TRPV1 expression were observed to have a poorer overall survival prognosis [21].

However, different results were reported in the study by Jiahui Lei et al. The article indicated that TRPV1 expression is higher in cervical cancer tissue compared to healthy tissue or tissue with epithelial dysplasia. Moreover, it was observed that high TRPV1 expression and low PTEN (phosphatase and tensin homolog) gene expression may serve as an independent prognostic factor in estimating patient survival time [6]. The same study also pointed to TRPV6 as a promising biomarker. The levels of mRNA and proteins were significantly lower in cells during the early stages of tumor development, suggesting that gene expression may correlate with tumor characteristics and patient prognosis. Patients with early-stage cervical cancer and low TRPV6 expression had a short progression-free survival time and overall survival [6,23]. Lastly, increased expression of the TRPM4 channel, which promotes cancer cell proliferation through the activation of the β-catenin-TCF/LEF pathway, was also linked to unfavorable prognosis [21,24].

#### 2.2.2. Calcium Release-Activated Calcium Channels

CRAC channels, particularly STIM1 and Orai1, play crucial roles in the progression of cervical cancer. Their elevated levels were observed in the majority of cervical cancer samples. STIM1 participates in processes such as cancer cell proliferation, migration, and angiogenesis. Its increased expression is associated with a higher risk of metastasis and reduced survival rate, while its inhibition suppresses cell proliferation, that combined, highlights its importance in tumor progression. STIM1 overexpression promotes cervical cancer cell invasion, whereas its knockout decreases its migratory potential [6]. In HPV-positive cervical cancer, STIM1 overexpression leads to increased VEGF-A (vascular endothelial growth factor) production, which facilitates cancer cell invasion [13]. That is why elevated STIM1 expression could serve as a valuable biomarker for cervical cancer.

### 2.3. Sodium Channels

#### 2.3.1. Voltage-Gated Sodium Channels

The voltage-gated sodium channels (VGSC) family consists of nine subtypes (Nav1.1–Nav1.9), encoded by the SCN1A–SCN11A genes [1,13]. In cervical cancer tissues, Nav1.2, Nav1.4, Nav1.6, and Nav1.7 were detected, with Nav1.2, Nav1.6, and Nav1.7 being particularly abundant, suggesting their potential as diagnostic markers. By contrast, only Nav1.4 was observed in healthy tissues. Further studies have revealed overexpression of Nav1.6 and Nav1.7b mRNA in HPV16-positive cervical cancer, accompanied by a diffuse distribution of the Nav1.6 protein across all layers of the squamous epithelium. Nav1.6 overexpression was specifically associated with the increased invasive potential of cancer cells [1,14]. Additionally, SCN8A overexpression in HeLa, SiHa, and CaSki cell lines increased MMP-2 activity, which promoted tumor invasion and progression. Inhibition of SCN8A significantly reduced the invasive potential of cancerous cells, pointing to the key role of VGSCs in tumor progression [13].

#### 2.3.2. Epithelial Sodium Channels

Epithelial sodium channels (ENaC) represent another family of sodium channels with potential prognostic significance in cervical cancer. Overexpression of ENaC was associated with favorable patient outcomes, particularly when SCNN1A, SCNN1B, and SCNN1G genes were simultaneously overexpressed. This phenomenon was more frequent in healthy tissues and low-grade cervical cancer, suggesting ENaC might act as a biomarker of good prognosis [14]. High ENaC expression may indicate less aggressive tumor characteristics, making it a promising tool for stratifying patients based on their risk.

Table 2 below presents a summary regarding ion channels as biomarkers

## 3. Mechanisms Linking Ion Channels to Cervical Cancer Metastasis

### 3.1. Role of Ion Channels in Cell Migration, Invasion, and Metastasis

#### 3.1.1. Migration

Cervical cancer cell migration is an essential prerequisite for metastasis. Several types of ion channels have been implicated in regulating cell motility.

Potassium (K^+^) Channels: Voltage-gated potassium channels, such as K_V_1.3 and K_V_10.1, are often upregulated in cancer cells. They are essential for maintaining the resting membrane potential and regulating calcium influx, which is necessary for cell contraction during migration. Inhibiting K^+^ channel activity reduces the migratory activity of cervical cancer cells [25]. K_Ca_3.1 channels also play a role, and inhibition of these channels leads to reduced invasion and migration [26].

Calcium (Ca^2+^) Channels: Calcium signaling is critical for various cellular functions, including cell migration. Voltage-gated calcium channels (VGCCs), such as Cav1.2, are important contributors to intracellular calcium levels. Dysregulation of VGCCs can result in increased calcium influx, promoting cytoskeletal remodeling and enhanced migration, as observed in some cervical cancer cell lines [27]. Calcium channel dysregulation in cancer cells leads to increased invasiveness and metastatic potential. Furthermore, calcium influx in stromal cells can alter their behavior, fostering an immunosuppressive TME that supports tumor growth [28].

Chloride (Cl^−^) Channels: Chloride channels, such as CLCN3 and CLCA2, are involved in cell volume regulation and are crucial for the formation of membrane protrusions during cell migration. Studies have shown that altered chloride channel activity can affect cell volume changes necessary for cell migration and invasion in cervical cancer [29]. Calcium-activated chloride channels (CaCl) open in response to an increase in cytosolic Ca^2+^. Numerous functions, such as afterdepolarization, transepithelial transport, vascular tone control, cellular excitability modulation, epithelial secretion, and cell proliferation in carcinogenesis processes, depend on these channels [30].

Sodium (Na^+^) Channels: Na_V_1.5 expression is significantly increased in cervical cancer tissues compared to normal tissues. Silencing Na_V_1.5 can lead to reduced cell migration and invasion, and this suggests its role in promoting metastatic behavior through activation of the ERK signaling pathway, which is crucial for cell motility [31]. Na_V_1.5 induces pro-migratory and pro-invasive properties. In particular, Na_V_1.5 activity causes the Na^+^-H^+^ exchanger type 1 (NHE-1) to undergo allosteric regulation, which raises its activity and causes the extracellular space to become more acidic. This favors the pH-dependent activity of proteolytic cysteine cathepsins [32].

Na_V_1.7 is upregulated in metastatic cervical cancer. Inhibition of Na_V_1.7 can result in decreasing cell proliferation and migration, implicating this channel in the metastatic cascade [33]. Na_V_1.7 may modulate intracellular calcium levels, affecting the cytoskeletal dynamics essential for cell movement [34]. Na_V_1.6 expression correlates with the advanced stages of cervical cancer. It is involved in boosting the activity of matrix metalloproteinases (MMP2) and Na^+^/H^+^ exchanger type 1 (NHE-1), further supporting the role of sodium channels in the metastatic process [35]. Increased sodium influx enhances membrane depolarization, facilitating cytoskeletal reorganization and promoting cellular motility.

#### 3.1.2. Invasion

Invasion involves the penetration of tumor cells into the surrounding tissue and ECM. Ion channels influence this process through the following aspects.

Regulating Matrix Metalloproteinase (MMP) Activity: MMPs are enzymes that degrade ECM components, facilitating tumor invasion. For instance, some ion channels influence the expression and activity of MMPs by modulating intracellular calcium levels. Activation of calcium channels, like Orai1, can lead to the downstream activation of transcription factors that promote MMP production, hence increasing invasion [36].

Controlling Cell Volume and Contractility: Cell volume and contractility changes are necessary for cells to navigate through the ECM. Ion channels, especially those involved in regulating water transport, such as aquaporins, indirectly contribute to invasion by facilitating cell deformation and movement through confined spaces. Furthermore, the activity of chloride channels and potassium channels contributes to cell shrinkage and swelling necessary for the invasive process [37].

Modulating Cell–ECM Interactions: Ion channels can affect the localization and turnover of focal adhesions through changes in intracellular calcium and pH, influencing the ability of cells to adhere to and detach from the ECM during invasion [16,38,39,40,41,42].

#### 3.1.3. Metastasis

Ultimately, metastasis is the culmination of migration and invasion, and ion channels play a vital role in the following processes.

Influencing Survival in Circulation: Ion channel dysregulation could contribute to increased resistance to anoikis (detachment-induced apoptosis), a phenomenon critical for tumor cell survival in circulation. Furthermore, some ion channels may contribute to the ability of cancer cell clusters to establish metastases, as has been shown with K_Ca_3.1 and the formation of multicellular spheroids [43].

### 3.2. Interaction Between Ion Channels and the Tumor Microenvironment

The tumor microenvironment (TME) refers to the complex environment surrounding a tumor, including various cell types, signaling molecules, blood vessels, and extracellular matrix components [44,45,46,47,48,49,50,51,52,53]. The TME plays a crucial role in cancer development, progression, and metastasis by influencing tumor cell behavior, immune response, and treatment outcomes [2]. The intricate relationship between ion channels and the tumor microenvironment has been a subject of growing interest in the field of cancer research. Tumor-associated immune cells, the immune response’s dysfunctional side, may arise as a result of the low pH and changes in the ionic composition of TME [17]. The activity of ion channels in all tumor tissue cells is significantly impacted by the acidity of inadequately perfused tumor regions [54].

Elevated concentrations of [K^+^] and [Na^+^] in the interstitium can cause acidification, severe hypoxia, and mechanical stress, which leads to cell necrosis. It also has an impact on immune cell infiltration [55,56]. As a result, the ionic composition of the tumor microenvironment is defined by components like protons that strongly influence channel activity, as well as by changed concentration gradients across the plasma membrane, which correspond to the changed electrochemical driving forces. Crucially, ion channels in stromal, immunological, and cancer cells detect the altered ionic composition and invariably alter their activity [19].

Altered expression of ion channels in tumor cells can affect their interactions with immune cells. For instance, changes in Ca^2+^ signaling can influence the ability of immune cells to recognize and eliminate tumor cells [57]. Cancer-associated fibroblasts (CAFs) are known to promote metastasis. There is evidence that ion channels, especially those responsive to mechanical forces, may influence the activity of CAFs and their capacity to remodel the ECM and facilitate cancer cell invasion [20].

As discussed earlier, ion channel activity can indirectly influence ECM remodeling by affecting the release of ECM-degrading enzymes, like MMPs. Moreover, changes in ion flux across cell membranes can lead to the altered activity of enzymes and proteins in the ECM, affecting its stiffness and topography, which in turn influences cell behavior [58]. Among the numerous effects generated by hypoxia, two—cell migration/invasion and resistance to apoptosis—are significantly associated with cell volume regulation, a complicated process meticulously governed by transporters and ion channels situated on the plasma membrane. Cells must prevent significant changes in volume, which threaten structural integrity and the stability of the intracellular environment. In reaction to hypotonic stress, cells protect themselves by initiating the outflow of osmolytes, including ions and certain organic molecules, together with water, to reduce the volume [59].

### 3.3. Ion Channel-Mediated Regulation of Cellular Adhesion and Motility

Cellular adhesion and motility are intricately linked in the metastatic process. Ion channels contribute to their regulation through several mechanisms. The formation and dissolution of focal adhesions are critical for cell migration. Calcium signaling, which is modulated by calcium channels, is essential for the regulation of focal adhesion assembly/disassembly and the activation of integrins, which are key for adhesion [6].

Intracellular calcium and pH levels, regulated by ion channels, influence cytoskeletal dynamics, which are essential for changes in cell shape and membrane protrusions during cell migration. Thus, ion channels can impact cell contractility, driving cell movement and invasion [60]. Ion channels can also indirectly influence cell–cell adhesion by regulating the expression and function of adhesion molecules, such as E-cadherin. For example, altered calcium signaling can disrupt cadherin-mediated cell adhesion, leading to increased cell motility [61].

## 4. Ion Channels as Therapeutic Targets

The past two decades of research have accumulated evidence that the association between specific ion channels and tumor progression will gradually become an important chapter of oncological treatment. The ion channel-oriented therapeutic approach offers a wide range of possible molecular targets, as the relationship between ion channels and carcinogenesis is multidimensionally complex and includes metabolic pathways, oxidative stress, immune response, and drug synergism. A multitude of cellular phenomena involved in ion channel-mediated neoplastic processes result in a diversity of the anticancer effects of ion channel-targeted therapies. Those effects include the promotion of apoptosis as well as the inhibition of cancer cell proliferation, migration, vascularization, and chemotherapy resistance [62]. Nevertheless, ion channels play important roles in nearly all physiological aspects of cell functionality, and thus, increasing cancer cell-specificity and avoiding adverse effects on healthy cells remains a challenge [63].

### 4.1. Potassium Channels as Therapeutic Targets

The application of potassium channel modulators constitutes a promising therapeutic approach in cervical cancer (CCa). On the one hand, potassium channel inhibitors may decrease the malignant properties of cancer cells; whereas, on the other hand, overexpression of some ion channels can sensitize neoplastic cells to specific cytotoxic drugs. The research on the most significant potassium channel types is presented in Table 3.

K_V_1.1 overexpression was reported in HeLa, SiHa, and C-33A CCa cell lines. Knockdown of this potassium channel decreases cellular levels of Wnt1 and Hedgehog (Hgh) in HeLa cells. It suppresses cell proliferation, migration, and invasion. In an animal model, the knockdown of K_V_1.1 results in smaller xenograft tumors in nude mice as well as their prolonged survival. According to clinical observations, higher expression of this ion channel correlates with poor prognosis and reduced survival time [64]. Migration of the HeLa cells is also suppressed under the influence of BDS-II, which causes K_V_3.4 channel blockage. BDS-II also increases PTEN activation and inactivates the AKT pathway [65]. K_V_10.1, also known as the ether-à-go-go-1 (EAG1) channel, is strongly associated with the proliferation of cancer cells, and thus, EAG1 inhibitors are suspected to have significant therapeutic potential. Among these inhibitors, tetrandrine reduces tumor growth in vivo [66]. In vitro, astemizole increases cancer cell apoptosis and decreases cell proliferation in HeLa, SiHa, CaSki, C-33A, and INBL cell lines [15]. In keratinocytes, EAG1 expression is conditioned by either the presence of HPV oncogenes or cellular immortalization. The combination of astemizole and imipramine decreases channel expression and increases apoptosis in E6/E7-transfected keratinocytes [1]. Calcitriol decreases EAG1 mRNA level and inhibits proliferation in the SiHa cell line [67].

Among intermediate-conductance-Ca^2+^ potassium channels, K_Ca_1.1 constitutes a potential CCa early marker, as higher immunostaining of the protein is more evident in high-grade dysplasia and cervical cancer tissues, which is of great importance, since detection of CCa in early stages constitutes the basis for effective treatment. What is more, in K14E7 transgenic mice, K_Ca_1.1-targeted estradiol treatment increases mRNA level and protein expression [69]. Following another study, activation of K_Ca_3.1 sensitizes cancer cells to the growth suppressant effects of DNA-binding fluorescent cation, Hoechst 33258 (H33258). Thus, K_Ca_3.1 channel activators could potentially be used to improve the penetration of small cationic toxins into CCa cells that express the discussed channel [70]. On the other hand, blocking K_Ca_3.1 with siRNA, as well as clotrimazole, results in inhibition of cell proliferation [71]; however, only siRNA blockage causes an increase in the proportion of apoptotic HeLa cells [71].

### 4.2. Sodium Channels as Therapeutic Targets

Sodium channels, widely known for their contribution to cell membrane depolarization in excitable cells, can also be found in non-excitable cells, where they participate in the regulation of proliferation and migration [80]. As sodium fluxes alter membrane potential, research examining the mutual influence of the discussed potential and cancer-associated factors would be of great relevance and significance. The most important types of sodium channels that were found overexpressed in CCa cells are presented in Table 3**.**

### 4.3. Calcium and TRP Channels

**Figure 2:**
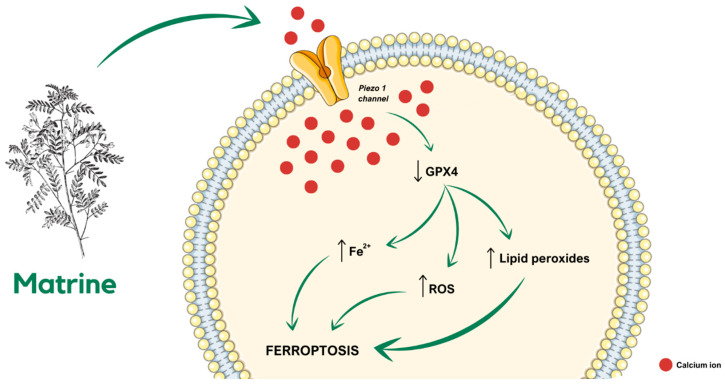
Schematic visualization of the chain of molecular events through which matrine causes ferroptosis in SiHa cells [79], (↑—increase; ↓—decrease).

Calcium channel regulation constitutes a vast area of molecular possibilities to inhibit CCa cell proliferation, as specific channel blockers or microRNAs could be administered in combination with fundamental anticancer therapy in order to enhance anti-CCa effectiveness. The most important types of calcium channels expressed in CCa are presented in Table 3. Matrine, an alkaloid found in plants from the *Sophora* genus, is widely known for its anti-CCa properties. According to the latest research, in a SiHa-derived tumor-bearing mouse model, treatment with matrine inhibits tumor growth in a ferroptosis-involved manner. Matrine administration causes a decrease in glutathione peroxidase 4 (GPX4) cellular level and, simultaneously, an increase in lipid peroxides, reactive oxygen species (ROS), and Fe^2+^ levels, eventually leading to cell death. Further investigation of the molecular complexity of the described dependencies revealed that ferroptosis is related to matrine-caused calcium influx through the Piezo1 channel (Figure 2), as Piezo1 small interfering RNA (siRNA) reverses the influence of matrine in the SiHa cells [79].

In CCa cell lines, calcium signaling alterations and disruptions occur commonly, and thus, aberrations in calcium channel expression are related to cancer prognosis [81]. Transient receptor potential vanilloid type 1 (TRPV1) is commonly overexpressed in CCa tissues in comparison to cervical intraepithelial neoplasia and normal epithelial tissue. Interestingly, TRPV1 expression is negatively correlated with phosphatase and tension homolog (PTEN) expression. Thus, the high TRPV1/low PTEN expression is considered a prognostic biomarker for overall CCa survival. At the cellular level, TRPV1 overexpression is associated with increased cell viability and colony formation and could be a biomarker for the chemoradiation response prediction. Transient receptor potential vanilloid subfamily member 6 (TRPV6) constitutes an element of the PRMT5/STC1/TRPV6/JNK axis. Trichostatin A (TSA) significantly suppresses TRPV6 level, as well as protein arginine methyltransferase 5 (PRMT5) level, simultaneously enhancing stanniocalcin 1 (STC1) and phosphorylated c-Jun N-terminal kinase (p-JNK) levels. In a described way, in the HeLa and CaSki cell lines, TSA suppresses cell proliferation and induces apoptosis and autophagy through regulation of the PRMT5/STC1/TRPV6/JNK axis [14].

Another widely overexpressed TRP channel in CCa is the transient receptor potential cation channel subfamily M member 4 (TRPM4) [1], which regulates the β-catenin pathway. In the HeLa cell line, the reduction of TRPM4 expression caused a decrease in cell proliferation. Accordingly, TRPM4 silencing caused a reduction of β-catenin/TCF/LEF-dependent transcription. On the contrary, TRPM4 overexpression in T-REx™ 293 cells increases the β-catenin level, leading to activation of the β-catenin/TCF/LEF pathway. The described chain of molecular events results in the facilitation of cell proliferation and invasion [82].

According to bioinformatic analyses, the transient receptor potential cation channel, subfamily M, member 7 (TRPM7) is correlated with varied types of microRNAs that modulate gene expression, such as miR-192-5p and miR-543 [77,78]. Overexpression of miR-192-5p in SiHa and CaSki cell lines reduces proliferation and migration, simultaneously facilitating apoptosis [77]. Similarly, miR-543 overexpression reduces proliferation and invasion in CCa cells [78]. TRPM7 overexpression decreases the tumor suppressive effects of miR-192-5p and miR-543 [77,78].

## 5. Innovative Diagnostic Tools Based on Ion Channel Activity

### 5.1. Development of Diagnostic Assays for Ion Channel Activity in Cervical Cancer

Evaluation of ion channel activity can be possible via diagnostic assays. Their development can offer valuable observation into cervical cancer progression and strengthen clinical decision-making. The above-mentioned assays may utilize various techniques, including fluorescence-based ion sensing, patch-clamp electrophysiology, and high-throughput screening platforms, to evaluate ion channel function in cervical cancer cell lines, patient-derived samples, or animal models [83]. Previous studies have shown that integrating ion channel-based diagnostic approaches with other clinicopathological variables, including tumor stage, HPV status, and patient demographics, allows a more comprehensive and accurate evaluation of cervical cancer risk and prognosis [84,85]. The potential of ion channels as diagnostic tools comes from their ability to serve as biomarkers for early detection, disease staging, and prognostic assessment of cervical cancer. The studies so far have emphasized the abnormal expression and dysregulation of various ion channels in cervical cancer tissues and cell lines, highlighting their promise as diagnostic and prognostic [86,87].

A recent study has identified the presence of functional voltage-gated sodium channels in primary cultures of human cervical cancer cells [41]. Using the whole-cell patch-clamp technique, the researchers were able to isolate and characterize a voltage-gated sodium current as the main component of the inward current in all the investigated cells. These findings suggest that voltage-gated sodium channels may play a crucial role in the pathogenesis of cervical cancer and could potentially serve as therapeutic targets or biomarkers for this disease [41].

Furthermore, high-throughput screening platforms have the potential to facilitate the discovery of novel ion channel modulators and their application in targeted cancer therapies. These automated screening systems enable the rapid evaluation of large chemical libraries, accelerating the identification of new pharmacological agents that can selectively modulate ion channel activity and potentially be developed into targeted cancer therapeutics [72,81].

### 5.2. Imaging Techniques and Biosensors for Ion Channel Detection

Investigators have directed their focus to utilize imaging techniques and biosensors to detect and monitor ion channel activity within cervical cancer cells. Molecular imaging techniques, including fluorescent biosensors, have been employed to map the localization and temporal dynamics of particular ion channels within the cervical tumor microenvironment [88]. The literature review shows that these methods facilitate real-time observation of ion channel activity, offering valuable insights into their involvement in the pathogenesis of cervical cancer. Electrochemical biosensors have emerged as a promising tool for the label-free detection and quantification of ion channels. They can measure changes in ion channel activity, which can serve as biomarkers for early cancer detection and disease monitoring [86,89,90]. Furthermore, those devices offer the ability to quantify ion channel activity and identify potential therapeutic targets [88,89,90].

Several studies have indicated that complex interactions between ion channels and diverse signaling cascades involved in cervical cancer, including those associated with vascular endothelial growth factor (VEGF) and its receptors, are significant for understanding the underlying molecular mechanisms [72,86,91]. The literature review shows that systems biology has been essential in understanding the complex regulatory pathways that control the development and progression of cervical cancer.

It has been established that utilizing these advanced imaging methods and biosensor technologies allows researchers to develop more effective diagnostic and therapeutic strategies that acquire an improved understanding of the role of ion channels in cervical cancer [41,92]. As has been previously reported in the literature, fluorescent imaging techniques have been utilized to monitor the activity of calcium channels, which are critical regulators of angiogenesis and tumor vascularization [86]. Furthermore, electrochemical biosensors have emerged as a valuable tool for the detection and quantification of ion channels, such as the sodium–boron cotransporter 1, a membrane-bound protein that plays a pivotal role in maintaining boron homeostasis [93]. Some studies have highlighted the promising applications of carbon dots, a class of fluorescent nanomaterials, in targeted cancer imaging and treatment [94,95]. These engineered nanoparticles can selectively localize within tumor tissues, providing a multifaceted platform for both diagnostic and therapeutic purposes [10,96,97].

### 5.3. Potential for Non-Invasive Diagnostic Techniques

Cervical cancer is a prevalent form of gynecological cancer, and early detection is crucial for effective treatment and management. Traditional diagnostic techniques, such as colposcopy and biopsy, may cause patient discomfort [98,99]. Prior research has investigated the potential of non-invasive diagnostic techniques, with particular attention to the role of ion channels in cervical cancer [87]. The abnormal expression and dysregulation of ion channels can contribute to the hallmarks of cancer, such as dysregulated proliferation, evasion of apoptosis, and enhanced metastasis [100,101].

A series of recent studies of ion channels in cervical cancer has prompted investigations into non-invasive diagnostic approaches, including liquid biopsy and wearable technology. A liquid biopsy, involving the analysis of circulating tumor cells, cell-free tumor DNA, or extracellular vesicles, may offer a non-invasive method to detect and monitor the presence of ion channel-associated biomarkers in cervical cancer patients [87,101,102]. Unfortunately, those non-invasive methods may encounter some difficulties before being applied to clinical use, due to their low sensitivity and specificity. Sensitivity ensures the accurate identification of individuals with cervical cancer, minimizing false-negative results, while specificity focuses on excluding those without the disease, reducing false-positive findings. Overcoming those challenges may be possible through advancements in analytical techniques and biomarker detection [4,103,104].

A better understanding of ion channel dysregulation seems to be the key to enhancing the detection of cervical cancer. The development of non-invasive diagnostic tools may also be crucial for risk evaluation and personalized treatment planning, ultimately leading to improved patient outcomes.

## 6. Combination Therapies Involving Ion Channel Modulation

### 6.1. Synergistic Effects of Ion Channel Modulators with Chemotherapy, Radiation, or Immunotherapy

Dysregulated ion channels significantly contribute to cancer cell chemoresistance by altering drug uptake and efflux, thereby reducing the effectiveness of chemotherapeutic agents. Ion channel modulators can be used in combination with more standard cancer therapies, such as chemotherapy, radiation, or immunotherapy, and have shown potential to improve these treatments. Ion channel modulators, for example, can sensitize cancer cells to certain chemotherapeutics, thereby enhancing their efficacy. Likewise, their conjugation with radiation therapy can enhance the radiation exposure damage to neoplastic cells causing an improved treatment efficacy [105]. For example, matrine has been shown to have an effect on the treatment of cervical cancer by promoting ferroptosis. Matrine was found to inhibit cancer cell proliferation with no apparent side effects. The treatment led to a reduction in GPX4 protein levels and an increase in lipid peroxides and Fe^2+^ content, indicating the induction of ferroptosis. Additionally, matrine upregulated the expression of the Piezo1 channel, facilitating calcium influx, and further promoting ferroptosis. These findings suggest that matrine exerts its anti-cancer effects by activating the Piezo1 channel and inducing ferroptosis, offering a promising therapeutic approach for cervical cancer [79].

Immunotherapy can also benefit from the inclusion of ion channel modulators [105,106]. Enhanced calcium signaling is closely linked to the development of tumors, promoting the malignant behaviors of cancer cells, activating various oncogenes and pathways, driving the creation and release of exosomes, and weakening the immune response by regulating immune checkpoints. Consequently, inhibiting calcium signaling could lower exosomal PD-L1 expression by reducing exosome secretion from cancer cells, presenting a promising strategy for cancer treatment through immune checkpoint blockade therapy. Therefore, all calcium-permeable ion channels and pumps emerge as natural therapeutic targets [107].

### 6.2. Potential for Ion Channel Inhibitors to Enhance Treatment Response

Na_v_ channels have been demonstrated to play a key role in glioblastoma multiforme (GBM) progression and stemness regulation, demonstrating a strong association between their expression and poor patient outcomes, particularly in the proneural subtype. A therapeutic strategy that combines Na_v_ channel inhibitors with standard treatments and repurposing antiepileptic drugs has shown significant potential for clinical application. These findings not only advance our understanding of the pathophysiology of GBM but also point to innovative approaches to more effectively treat this aggressive cancer [108]. Moreover, the voltage-gated sodium channel modulator DPI-201-106 has been shown to reduce glioblastoma cell viability by inducing cell cycle arrest and apoptosis [95]. Similar approaches can be applied to cervical cancer, where a transient receptor potential vanilloid 1 (TRPV1) inhibitor may enhance the efficacy of existing treatments and potentially overcome resistance to cisplatin therapy [96]. The combination of ion channel inhibitors with DNA damage response inhibitors has demonstrated synergistic effects in preclinical models, suggesting that these inhibitors can be effectively integrated into combination therapy strategies [95].

Researchers have demonstrated that K_ATP_ channels are active in HPV-positive cells and necessary for HPV oncoprotein expression. The regulatory subunit SUR1 of the K_ATP_ channel complex was upregulated in HPV-positive cervical cancer cells and patient samples, dependent on the E7 oncoprotein. Inhibition or knockdown of SUR1 significantly reduced cell proliferation by inducing G1 cell cycle arrest, confirmed in both in vitro and in vivo assays. The study suggests that K_ATP_ channels drive cell proliferation through the MAPK/AP-1 signaling pathway. These findings highlight the potential of targeting KATP channels with clinically available inhibitors as a novel therapy for HPV-driven cervical cancer [10].

Nifedipine has been shown to reduce PD-L1 expression in colorectal cancer cells and PD-1 expression in CD8+ T cells, thereby reactivating immune surveillance and potentially enhancing the efficacy of PD-1-based immunotherapy [97]. Similarly, calcium channel blockers, such as amlodipine, felodipine, manidipine, and cilnidipine, have demonstrated significant efficacy in inhibiting filopodia formation, directional cell migration, and invasive behavior in breast and pancreatic cancers [91,98]. Notably, amlodipine selectively induces the autophagic degradation of PD-L1 in a calcium-dependent manner, reducing PD-1 binding, increasing T-cell cytotoxicity, suppressing tumor growth, and improving the effectiveness of anti-PD-L1 therapy in preclinical models [99]. Furthermore, the T-type calcium channel blocker KTt-45 has been identified as a potential anticancer agent with promising efficacy against HeLa cervical cancer cells. As can be seen from the results of the cited study, KTt-45 has cytotoxic activity on numerous cancer cell lines, and it is very specific for HeLa cells. Its mechanism involves inducing mitochondrial-dependent apoptosis, as evidenced by cytoplasmic vacuole accumulation, chromatin condensation, nuclear fragmentation, and the activation of caspase-9 and caspase-3 [100]. Uncovering the full anticancer therapeutic potential of calcium channel antagonists requires further research.

A fact worth noting is that certain ion channels play a critical role in cancer cell migration and invasion, making them attractive targets for reducing metastatic potential [101,102]. The mechanosensitive ion channel Piezo1 has been identified as a potential prognostic and therapeutic target in cervical cancer. Elevated expression of Piezo1 was observed in cervical cancer tissues and cells, particularly in patients with lymph node metastasis. Silencing Piezo1 significantly reduced cervical cancer cell invasion and migration, while activation with the selective agonist enhanced these processes. It was found that activation of Piezo1 is capable of regulating the release of extracellular ATP, which can enhance invasion, migration, and pseudopodium formation in cervical cancer. The in vivo analysis also strengthened the findings on the importance of Piezo1 in tumor progression. The results of the cited study suggest that Piezo1 contributes to cervical cancer progression by facilitating ATP release and could serve as a promising target for therapeutic and prognostic applications [4].

Ion channels play a major role in modulating the tumor microenvironment, influencing factors like pH and hypoxia that are closely associated with treatment resistance. Targeting these channels can potentially improve the effectiveness of already approved therapies [103]. Recent studies have also found a close correlation between high sodium concentrations within the tumor microenvironment and adverse clinical outcomes, with several types of Na^+^ channels acting as important regulatory factors. These novel findings open the door for therapeutic innovation, which is already being exploited in the clinic as reflected in ongoing clinical trials aimed at targeting this altered sodium microenvironment [104].

### 6.3. Clinical Trials and Studies Exploring Combination Strategies

A venom-derived peptide, SOR-C13, has been identified as a potent TRPV6 blocker, effectively inhibiting tumor growth in ovarian and breast cancer models. Following successful preclinical studies and safety validation, SOR-C13 entered phase 1 clinical trials, demonstrating safety, tolerability, and anti-tumor activity, particularly in pancreatic cancer. Encouraged by these results, Phase 1b trials are underway, and SOR-C13 has been granted orphan drug status by the FDA for ovarian and pancreatic cancer treatment [105].

Chemotherapy-induced peripheral neuropathy, a common side effect of drugs like oxaliplatin, often causes hypersensitivity with few effective treatments. TRPA1, an ion channel linked to chemotherapy-induced peripheral neuropathy, is modulated by the Sigma-1 receptor. Sigma-1 receptor antagonists disrupt this interaction, reducing TRPA1 activity and preventing painful symptoms in a mouse model of oxaliplatin neuropathy. These findings highlight the Sigma-1 receptor antagonists as a potential strategy for treating chemotherapy-induced peripheral neuropathy and neuropathic pain [106].

The presence of the Kir6.2/SUR2 potassium channel could be a positive indicator for the prognosis of gynecologic cancers. Using minoxidil to stimulate this channel effectively halts tumor growth in a xenograft model of ovarian cancer. Minoxidil changes the metabolic and oxidative state of cancer cells, leading to mitochondrial disruption and significant DNA damage, which triggers a cell death pathway independent of caspase-3. These findings suggest that repurposing FDA-approved K^+^ channel activators could offer a novel, safe adjuvant therapeutic approach to traditional chemotherapy for treating gynecologic cancers [107].

The phytochemical α-mangostin exhibits powerful antineoplastic effects, making it a promising candidate to use as an adjunct to standard therapies for the treatment of cervical neoplasia. It potently inhibits cell proliferation in a dose-dependent fashion in cell lines containing increased HPV16 copy numbers. Notably, α-mangostin greatly reduces the expression of HPV oncogenes E6 and E7 as well as the KCNH1 gene both in vitro and in vivo. Additionally, it modulates cytokine profiles, decreases Ki-67 levels, and suppresses tumor growth in xenografted mice. These findings underscore the potential of α-mangostin as a therapeutic agent for both the prevention and treatment of cervical cancer [108].

## 7. Challenges and Future Directions

Ion channels are essential for many cell functions. Their distribution varies depending on the cell type and disease stage. So far, scientists have been able to identify several profiles of ion channels that correlate with tumor behavior, drug resistance, and clinical outcome. This makes them promising targets for cancer detection and therapy. However, there are still many challenges. Ion channels are responsible for the regulation of many processes, making it hard to isolate their influence on cancer progression. We also need a more profound understanding to fully distinguish tumor-promoting from tumor-suppressing effects.

Another challenge is the lack of selectivity of the discussed proteins. Despite some ion channels being recognized as potential biomarkers, their specificity, and reliability in identifying cervical cancer cells are limited. Moreover, the expression patterns of ion channels vary significantly among cervical cancer patients. This restricts the determination of common biomarkers and treatment targets. It also leads to different treatment outcomes. This shows a need for more clinical trials in order to validate the efficacy, safety, and optimal use of the discussed methods. Additionally, the development of standardized assays to measure ion channel activity in clinical samples is essential. While promising, ion channel-based diagnostic tools require refinement to achieve clinical-grade sensitivity, specificity, and cost-effectiveness. Innovative diagnostic tools should be further developed and validated for the proper monitoring of cervical cancer progress. The development of biosensors and imaging agents that target ion channels could improve early detection, particularly when combined with other diagnostic workflows.

Due to the complexity of ion channel physiology, resistance to treatment may occur. Cells may display compensatory pathways or activation of alternative ion channels. Moreover, the presence of ion channels in every cell increases the risk of targeting healthy tissues, which may lead to unexpected complications. The integration of genomics, transcriptomics, and proteomics data may be crucial in the development of more selective modulators. Advances in molecular modeling, drug design, and nano-technology-based delivery systems may play a key role in creating more personalized medicine. For such tailored therapies, we also need patient-specific data, such as ion channel expression profiles and genetic mutations. This shows the great importance of creating accurate predictive models and biomarker-driven clinical trials.

Ion channel modulators have the potential to be used in combination therapies to enhance the effectiveness of conventional treatments, such as chemotherapy, radiotherapy, and immunotherapy. It may also help in overcoming the resistance of the cancer cells. To optimize these effects, more research into synergistic effects and optimal sequencing is required. Conducting large-scale, longitudinal studies across diverse populations is critical to validate the clinical utility of ion channel biomarkers and therapies. Such studies can also uncover regional and demographic differences in ion channel-related cancer biology.

## 8. Conclusions

Despite significant advances in understanding the factors contributing to the development of cervical cancer, as well as the implementation of preventive actions, such as HPV vaccination and numerous screening programs, this cancer remains a significant epidemiological, diagnostic, and therapeutic challenge. Therefore, it is crucial to conduct advanced research, including molecular diagnostics, which will not only deepen our understanding of the disease’s pathogenesis but enable us to develop modern diagnostic methods and effective targeted therapies.

Ion channels seem to be promising targets for such research, due to their critical role in the progression of cervical cancer. Their diverse functions, including influencing the processes of cancer cell proliferation, migration, and invasion, make them important potential biomarkers for cancer detection and disease progression assessment. Additionally, the evaluation of gene expression may be significant in predicting responses to traditional anticancer treatments and determining patient prognosis.

Advances made in both in vivo studies and animal models have shown promising results. However, challenges such as the complexity of ion channel interactions and the risk of adverse effects still require extensive research. Diagnostic methods under development also need further refinement to ensure the highest sensitivity and specificity, as well as the ability to detect cancer in its early stages. To meet these goals, future work must rigorously address current limitations—small patient cohorts, model heterogeneity, off-target safety concerns, and drug-delivery hurdles—so that the most promising ion-channel targets can advance from proof-of-concept to well-designed clinical trials and, ultimately, everyday clinical practice. Thus, continued research will be crucial to fully harness the potential of ion channels in cervical cancer diagnostics and treatment.

## Figures and Tables

**Table 1:** Types of ion channels associated with cervical cancer.

Types of Ion Channels	Ion Channels Characteristics	Ref.
Voltage-gated Ion Channels	Activated by changes in membrane potential. These channels are critical for the generation and propagation of electrical signals in neurons and muscle cells. In cervical cancer, the altered expression or function of voltage-gated sodium and potassium channels can influence cell proliferation and metastatic potential.	[1,2]
Ligand-gated Ion Channels	Open in response to the binding of specific molecules, such as neurotransmitters or hormones. This class includes channels that respond to ligands relevant to cancer biology, such as growth factors. Specific ligand-gated channels, such as P2X7R, can facilitate calcium influx, which may promote oncogenic signaling pathways in cervical cancer cells.	[3]
Mechanosensitive Ion Channels	Respond to mechanical stimuli, such as stretching or pressure changes. They play a role in tumor microenvironment interactions, where mechanical forces, including growth and migration, can influence cell behavior. In cervical cancer, mechanosensitive channels may contribute to the tumor’s response to changes in tissue stiffness and extracellular matrix remodeling.	[4]
Leakage Ion Channels	Allow for the constant transportation of ions across the membrane, which is critical for maintaining the resting membrane potential. Sodium and potassium leakage channels are crucial in maintaining the electrical activity of cervical cancer cells, which might influence their growth and migratory patterns.	[5]
Calcium Channels	Allow the influx of calcium ions into the cell. Elevated intracellular calcium levels activate various signaling pathways associated with cancer progression, including those involved in cell growth, differentiation, and apoptosis. In cervical cancer, aberrant calcium signaling has been linked to enhanced cell survival and resistance to therapy.	[6]
Chloride Channels	Facilitate the transport of chloride ions across the cell membrane. These channels are involved in maintaining cell volume and regulating electrical excitability. In cervical cancer, alterations in chloride channel activity can influence tumor cell migration and invasion capabilities. CLCN3 has been associated with tumor metastasis and prognosis in cervical cancer.	[7,8]
Transient Receptor Potential Channels	Involved in various cellular functions, including calcium influx. They are studied to be prognostic tools and therapeutic targets.	[9]
ATP-Sensitive Potassium Channels	Have been associated with human papillomavirus (HPV). They can be considered as new therapeutic targets.	[10]

**Table 2:** Ion-channel biomarkers in cervical cancer—expression patterns, functional impact, and their diagnostic or prognostic usability (↑—increase; ↓—decrease;→—determines).

Ion-Channel (Gene)	Reported Expression Pattern in CCa	Clinical/Biological Significance	Biomarker Role
KV1.1 (KCNA1)	↑ mRNA and protein in tumors; higher levels - shorter survival	Drives proliferation, migration, and invasion	Prognostic (poor)
KV10.1 (KCNH1/Eag1)	Markedly over-expressed vs. normal cervix	Correlates with tumor growth and possible metastasis	Diagnostic/prognostic
KCa1.1 (KCNMA1)	Detected from LSIL, peaks in HSIL/CCa	Tracks dysplasia progression	Early diagnostic
KCa3.1 (KCNN4)	↑ in CCa tissue and HeLa cells; level rises with grade	Supports proliferation	Diagnostic
Kir6.2 + SUR1 (K-ATP)	Absent in healthy cervix; high in stage IV and invasive tumors	HPV-E7 up-regulates ABCC8/SUR1; linked to MAPK/AP-1	Progression marker
TRPC6	↑ expression associates with lymph-vascular invasion	Promotes invasive behavior	Progression/prognostic
TRPV1	Conflicting data: some studies ↓, others ↑ with low PTEN	Altered levels predict overall survival and therapy response	prognostic
TRPV6	↓ mRNA/protein in early-stage SCC; low levels determine shorter PFS/OS	Regulates Ca^2+^ signaling and apoptosis	Prognostic (poor)
TRPM4	Overexpressed; activates β-catenin pathway	Enhances proliferation → worse prognosis	Prognostic (poor)
STIM1 (CRAC)	↑ in most CCa samples; high levels determine metastasis, ↓ survival	directs Ca^2+^ influx, VEGF-A release	Prognostic
NaV1.6 (SCN8A)	↑ mRNA/protein in HPV16^+^ CCa, diffuse epithelial staining	Boosts MMP-2, invasiveness	Diagnostic/prognostic
NaV1.7 (SCN9A)	Frequently upregulated in metastatic CCa	supports migration and invasion	Prognostic

**Table 3:** The summarization of research targeting ion channels in CCa treatment and their outcomes regarding in vitro studies, animal models, and clinical observations.

Channel Type	In Vitro Studies	Animal Models	Clinical Observations	Ref.
K^+^ channels				
K_V_1.1	In HeLa cells, the knockdown of K_V_1.1 decreases cellular levels of Wnt1 and Hgh. It suppresses cell proliferation, migration, and invasion.	Knockdown of K_V_1.1 results in smaller xenograft tumors in nude mice as well as their prolonged survival.	Higher expression of K_V_1.1 correlates with poor prognosis.	[64]
K_V_3.4	BDS-II, a K_V_3 channel blocker, causes inactivation of the AKT pathway and inhibition of cell migration in HeLa cells.			[65]
K_V_10.1	Treatment with astemizole results in decreased proliferation and increased apoptosis in HeLa, SiHa, CaSki, C-33A, and INBL cell lines. The combination of astemizole and imipramine decreases channel expression and increases apoptosis in E6/E7-transfected keratinocytes. Treatment with calcitriol causes a decrease in mRNA level and proliferation in the SiHa cell line.	Treatment with tetrandrine results in inhibition of tumor growth in xenograft mice. In transgenic mice, K14E7 is treated with estrogens, and increased mRNA and protein expression is observed in CCa tissues.	Higher expression of K_V_10.1 is observed in high-grade cervical lesions compared to low-grade lesions and normal tissues.	[1,15,66,67,68]
K_Ca_1.1		In K14E7 transgenic mice with CCa, estradiol administration increases mRNA level and protein expression.	Potential early CCa marker; higher immunostaining of the protein is more evident in high-grade dysplasia and cervical cancer tissues.	[69]
K_Ca_3.1	siRNA-induced downregulation increases apoptosis in HeLa cells. Clotrimazole reduces mRNA expression in the HeLa cell line.			[70,71,72]
K_ir_6.2	Glibenclamide decreases cell proliferation in HeLa cells.		Compared to non-invasive tumors and normal tissues, K_ir_6.2 expression is significantly higher in invasive tumors.	[5]
Na^+^ channels				
Na_V_1.2	In primary cultures transfected with E7 oncogene, high mRNA expression is observed.		In cancerous biopsies, mRNA expression is higher than in normal biopsies.	[13]
Na_V_1.6	In primary cultures, mRNA overexpression promotes invasiveness, which is mediated by MMP2 activity.		In CCa biopsies, protein pattern expression is more extensive than in normal biopsies.	[73,74]
Na_V_1.7	In primary cultures, mRNA overexpression is observed.		In CCa biopsies, protein pattern expression is more extensive than in normal biopsies.	[73]
Ca^2+^ and TRP channels				
TRPV1	TRPV1 overexpression is associated with increased cell viability and colony formation and thus could be a biomarker for the chemoradiation response prediction.		In CCa tissues, TRPV1 expression is significantly higher than in cervical intraepithelial neoplasia and normal epithelial tissues.	[75]
TRPV6	In HeLa and CaSki cell lines TSA suppresses CCa cell proliferation and induces apoptosis and autophagy through regulation of the PRMT5/STC1/TRPV6/JNK axis.		In early-stage squamous cell CCa, reduced expression is correlated with poor prognosis.	[14,76]
TRPM4			In CCa specimens, overexpression is observed in gene sequence analysis.	
TRPM7	TRPM7 overexpression decreases tumor suppressive effects of miR-192-5p and miR-543.			[77,78]
Piezo1	Matrine administration causes a decrease of GPX4 cellular level and an increase of lipid peroxides, ROS, and Fe^2+^ levels, eventually leading to cell death.	In the SiHa-derived tumor-bearing mouse model, treatment with matrine inhibits tumor growth in a ferroptosis-involved manner.		[79]
